# Using 4D dose accumulation to calculate organ‐at‐risk dose deviations from motion‐synchronized liver and lung tomotherapy treatments

**DOI:** 10.1002/acm2.13627

**Published:** 2022-04-29

**Authors:** William S. Ferris, Edward H. Chao, Jennifer B. Smilowitz, Randall J. Kimple, John E. Bayouth, Wesley S. Culberson

**Affiliations:** ^1^ Department of Medical Physics School of Medicine and Public Health University of Wisconsin–Madison Madison Wisconsin USA; ^2^ Accuray Inc. Madison Wisconsin USA; ^3^ Department of Human Oncology School of Medicine and Public Health University of Wisconsin–Madison Madison Wisconsin USA; ^4^ University of Wisconsin Carbone Cancer Center, University of Wisconsin–Madison Madison Wisconsin USA

**Keywords:** Radixact, Synchrony, tomotherapy

## Abstract

Tracking systems such as Radixact Synchrony change the planned delivery of radiation during treatment to follow the target. This is typically achieved without considering the location changes of organs at risk (OARs). The goal of this work was to develop a novel 4D dose accumulation framework to quantify OAR dose deviations due to the motion and tracked treatment. The framework obtains deformation information and the target motion pattern from a four‐dimensional computed tomography dataset. The helical tomotherapy treatment plan is split into 10 plans and motion correction is applied separately to the jaw pattern and multi‐leaf collimator (MLC) sinogram for each phase based on the location of the target in each phase. Deformable image registration (DIR) is calculated from each phase to the references phase using a commercial algorithm, and doses are accumulated according to the DIR. The effect of motion synchronization on OAR dose was analyzed for five lung and five liver subjects by comparing planned versus synchrony‐accumulated dose. The motion was compensated by an average of 1.6 cm of jaw sway and by an average of 5.7% of leaf openings modified, indicating that most of the motion compensation was from jaw sway and not MLC changes. OAR dose deviations as large as 19 Gy were observed, and for all 10 cases, dose deviations greater than 7 Gy were observed. Target dose remained relatively constant (D95% within 3 Gy), confirming that motion‐synchronization achieved the goal of maintaining target dose. Dose deviations provided by the framework can be leveraged during the treatment planning process by identifying cases where OAR doses may change significantly from their planned values with respect to the critical constraints. The framework is specific to synchronized helical tomotherapy treatments, but the OAR dose deviations apply to any real‐time tracking technique that does not consider location changes of OARs.

## 1 INTRODUCTION

Synchrony is a real‐time motion tracking system for helical tomotherapy treatments available on the Radixact linear accelerator (Accuray Inc., Sunnyvale, CA).^1^ This system uses light‐emitting diodes placed on the patient's chest and kilovoltage (kV) images acquired perpendicular to the megavoltage (MV) therapeutic beam to monitor motion. The collimation is adjusted in real‐time to synchronize the delivery of the radiation with the respiration‐induced movement of the target. (Synchrony can also be used for non‐respiratory motion that will not be discussed in this work.) The binary 64‐leaf multi‐leaf collimator (MLC) corrects for motion in the IEC‐*X* and IEC‐*Z* directions (axial plane) by shifting the leaf openings. The jaws correct for motion in the IEC‐*Y* direction (superior/inferior [sup/inf]) by continuously swaying.

The goal of the tracking is to keep target dose the same as planned despite the motion. However, most motion tracking systems such as Synchrony do not consider the location and shape changes of organs at risk (OARs) when compensating the treatment. Therefore, a method to calculate OAR dose deviations after motion correction is of interest. In motion gating systems, the planned treatment is always delivered with the patient at the same respiratory phase. In this case, OAR doses can be more accurately estimated with the original 3D treatment plan because OARs are in a similar position during that specific respiratory phase. OAR doses are not as easily calculated for tracking treatments as the patient is treated at all phases of the respiratory cycle.

There have been several studies demonstrating Synchrony's ability to accurately correct the treatment for target motion.^1^, ^2^, ^3^, ^4^, ^5^, ^6^, ^7^ However, there have not been any publications to date investigating the effect of the motion compensation on doses to OARs, which is a critical component of successful tracking since the planned treatment is changed in real time.^8^ In fact, to our knowledge there are no works in the literature investigating the effect of any real‐time tracking system on dose deviations to OARs.

Calculating dose to OARs from tracking treatments requires deformation information as OARs typically do not move rigidly with the target, especially for treatment sites in the thorax or abdomen.^9^ The deformation information is commonly obtained using four‐dimensional computed tomography (4DCT) datasets. Methods of dose accumulation with 4DCT datasets have been published in the literature,^10^, ^11^ but not applied to Radixact Synchrony deliveries or used to calculate OAR dose deviations. Chao et al. used rigid shifts of a 3D image and a motion‐compensated delivery plan (MLC and jaws) to calculate dose delivered to a phantom from Synchrony deliveries.^2^ However, these calculations did not account for deformations and were only demonstrated with phantom volumes. Zhang et al. incorporated respiratory motion into helical tomotherapy deliveries using a 4DCT set, but the calculations were for breathing‐synchronized treatments (i.e., building motion into the treatment plan and coaching the patient to breath the same during treatment) and not real‐time motion‐synchronized treatments.^12^, ^13^

The goal of this work was to develop a novel, in‐house 4D dose accumulation framework capable of calculating doses from motion‐synchronized helical tomotherapy treatments and to use the framework to calculate dose deviations to OARs for lung and liver subjects. The framework is specific to synchronized helical tomotherapy treatments, but the results on OAR dose deviations apply to any real‐time tracking treatment techniques.

## 2 METHODS

The framework was used to calculate dose deviations for five lung and five liver stereotactic body radiation therapy (SBRT) patients previously treated at our institution with non‐Synchrony respiratory management. The specifics of the workflow may change for other institutions as protocols, software, and data storage types may differ. However, the general concept of the workflow will remain the same. This work focuses on lung SBRT treatments as these often necessitate intrafraction respiratory motion management.^9^ Key steps of the framework include the following:

1. Obtain input data: 4DCT images and helical tomotherapy treatment plan.
2. Get 3D location of the target on each phase of the 4DCT.
3. Assume respiratory phase pattern from mathematical function or chest amplitude data.
4. Split treatment plan into 10 plans based on respiratory phase pattern.
5. Apply MLC corrections to each of the 10 plans.
6. Apply jaw corrections to each of the 10 plans.
7. Calculate dose for each plan on corresponding 4DCT phase.
8. Calculate deformable image registration (DIR) between each phase and reference phase.
9. Map the dose to the reference phase using DIR.
10. Sum doses to create Synchrony‐accumulated dose.
11. Compare Synchrony‐accumulated dose to static planned dose.

### 2.1 Treatment planning

The inputs to the framework are a helical tomotherapy treatment plan and a 4DCT dataset. The treatment plan is a plan that is intended to be treated with Synchrony and is planned on one of the phases of the 4DCT scan, designated as the reference phase. The reference phase is often the maximum inspiration or exhalation phase; however, using a mid‐ventilation phase will maximize the range of motion that can be corrected^4^ and reduces the impact of the unflattened beam profile on the output as the target moves off axis.^7^ The reference phase used for the calculations should be replicated by the patient during the pre‐treatment alignment images (e.g., chest/abdomen/diaphragm position and lung volume).

Patient selection was performed using the recommendations from Chen et al., including a diameter of solid tumor 2–8 cm, magnitude of tumor motion in sup/inf direction less than 4 cm, and high rigidity of the target.^6^ Characteristics of the treatments in this work are shown in Table 1. The 4DCT scans were acquired using a Siemens Somatom Edge scanner (Siemens AG, Munich, Germany). The Siemens scanner acquires helical projection data in 250 ms of X‐ray tube rotation using constant couch speed with a pitch of ∼0.1 and bins the reconstructed images into 10 CT datasets. The 10 datasets will be referred to consistent with the vendors nomenclature: inhalation 20%, 40%, 60%, 80%, 100%, and exhalation 80%, 60%, 40%, 20%, and 0%.^14^

**TABLE 1:** Details of each clinical case used in this work

Subject	Jaw (cm)	Prescription	Target location	PTV vol (cm^3^)	BOT (s)
Lung 1	2.5	50 Gy to PTV D98%	Medial posterior RML	12.2	576.5
Lung 2	2.5	50 Gy to PTV D98%	Posterior RSL	129.5	1060.3
Lung 3	1	50 Gy to PTV D98%	Lateral RML	30.6	1771.9
Lung 4	2.5	50 Gy to PTV D98%	Medial RIL	26.2	418.0
Lung 5	1	50 Gy to PTV D98%	Posterior RIL	38.5	1290.7
Liver 1	2.5	40 Gy to PTV D95%	Inferior/anterior liver	149.1	336.3
Liver 2	1	40 Gy to PTV D95%	Inferior/posterior liver	68.2	1297.8
Liver 3	2.5	55 Gy to PTV D95%	Superior liver	92.8	548.5
Liver 4	2.5	50 Gy to PTV D95%	Superior/anterior liver	76.5	653.4
Liver 5	1	50 Gy to PTV D95%	Inferior/anterior liver	33.8	1047.7

All treatments were five fractions.

Abbreviations: BOT, beam‐on time; RIL, right inferior lobe; RML, right middle lobe; RSL, right superior lobe.

Clinically, the subjects included in this work were treated with tight margins (3–5 mm) using a gated delivery. For this work, they were replanned for helical tomotherapy delivery on Radixact using 5‐mm gross tumor volume (GTV) to planning target volume (PTV) margins and the same clinical objectives as their original treatment plan. The prescriptions for each subject are shown in Table 1. The plans were evaluated with typical metrics used for evaluating SBRT lung and liver treatments at our institution.^15^, ^16^ The clinical structure sets used for treatment were transferred to the reference phase of the 4DCT using DIR calculated in MIM (MIM Software Inc., Cleveland, OH), verified, and modified, if necessary, before planning the tomotherapy treatment. DIR will be described in Section 2.5. Additional planning structures unique to helical tomotherapy were added if necessary. The choice of jaw setting for each plan was influenced by the magnitude of motion observed on the 4DCT. The maximum ranges of motion that can be compensated with the 1‐ and 2.5‐cm jaws are 4 and 2.5 cm, respectively.^4^ For example, the Lung 3 subject had a motion amplitude of 2.8 cm in the sup/inf direction therefore could only be treated with the 1‐cm jaw.

### 2.2 Simulating the target motion pattern

**FIGURE 1:**
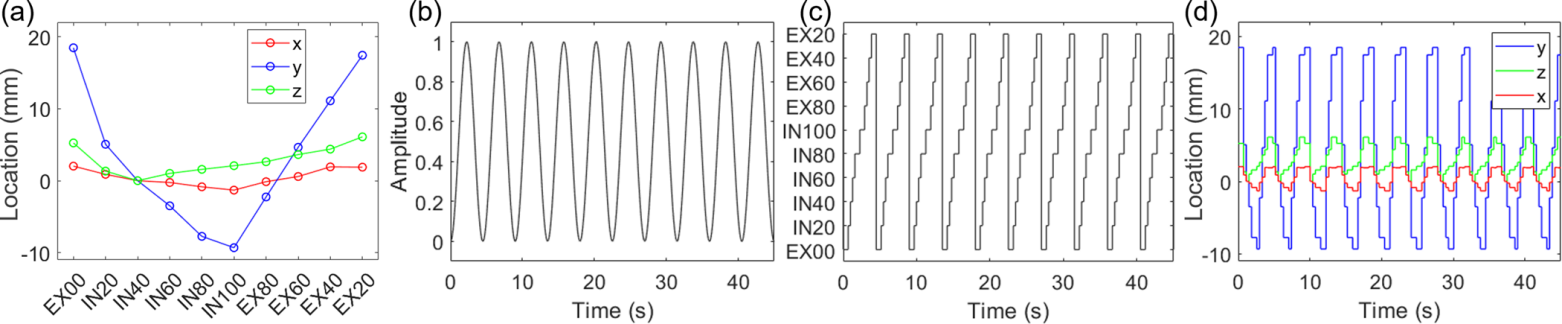
(a) Location of the target centroid in each phase relative to the reference phase derived from the 4DCT images for Lung 3. (b) The continuous respiratory amplitude pattern. (c) The discrete respiratory pattern mapped to each of the 10 phases. (d) The discrete mathematical target motion pattern. 4DCT, four‐dimensional computed tomography

The motion compensated treatment plan was generated using the target location at every point during the treatment. The target location was obtained by contouring the target on the reference phase, transferring the target to the other phases with DIR, and obtaining the centroid of the contour on each phase. Figure 1 shows an example of the target locations relative to the reference phase for Lung 3. The discretization of the target motion pattern is necessary to maintain consistency with the 10 discrete locations of the target centroid on the 10 4DCT phases. The temporal pattern of respiration was derived using a mathematical motion pattern (e.g., sin, sin^2^ *^n^*).^17^ A patient‐specific pattern of respiration could theoretically be used instead of a generalized motion pattern. However, these patterns are not trivial to obtain and may change between simulation and treatment or between treatment fractions. Also, the pattern of respiration only changes the relative weighting of the sinogram projections among the 10 phases but does not change the location of the target in each phase and therefore does not change the jaw sway or MLC shifts. The period of respiration was chosen to be 4.5 s for this work based on typical respiration characteristics.^8^, ^9^ However, the period of respiration can be changed based on patient‐specific characteristics if appropriate. The mathematical motion pattern was used to assign each time point to 1 of the 10 phases, then the 3D location of the target in each phase was assigned to that time point. This is demonstrated for one case in Figure 1.

### 2.3 Splitting the treatment plan into each phase

**FIGURE 2:**
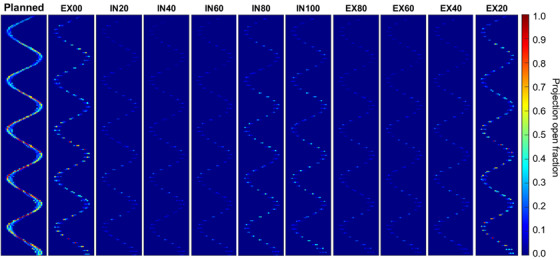
Example of assigning sinogram projections from the original treatment plan to each phase of the 4DCT for Lung 3. The central 30 MLC leaves and the first 250 projections of the treatment are shown. 4DCT, four‐dimensional computed tomography; MLC, multi‐leaf collimator

The next step was to split the original planned sinogram into 10 sinograms, shown in Figure 2. The treatment is divided into 51 projections per gantry rotation, and each projection is divided into 3 sub‐projections for dose calculation. Sinogram splitting was performed by assigning every sub‐projection from the original sinogram to a new sinogram corresponding to each phase, while conserving leaf open time. A sub‐projection can be assigned to multiple phases if the patient is in multiple respiratory phases during that projection time. In Figure 2, red is mapped to a value of unity and indicates that the MLC leaf is open for the entire projection time. The relative brightness of each sinogram is primarily determined by relative time spent in that phase; therefore, the sinograms at peaks of inhale and ends of exhale are generally brighter than the mid‐respiration phases. The individual sinograms sum to the planned sinogram such that total brightness (or leaf open time) is conserved.

### 2.4 Motion‐synchronized treatment plans

The next step was to apply motion corrections to the planned collimation. The corrections were calculated using software from the vendor that contains the motion‐tracking algorithm. Information about the algorithm is provided by Schnarr et al. or can be found in the Radixact Physics Essentials Guide.^1^, ^18^

**FIGURE 3:**
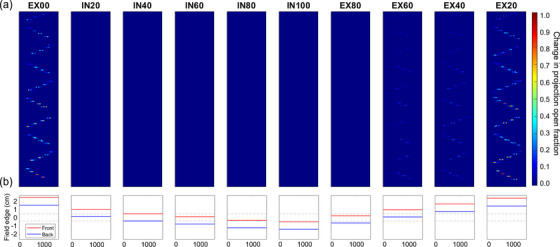
Example of generation of motion‐synchronized treatment plans for Lung 3. (a) Differential sinograms expressed as the absolute difference between the original MLC sinogram and the motion‐synchronized sinogram. The central 30 MLC leaves and the first 250 projections of the treatment are shown. (b) Jaw field edges after compensation projected to isocenter for each phase as a function of projection in the treatment. The dotted gray lines indicate the centered jaw positions. MLC, multi‐leaf collimator

Motion corrections were applied separately for the MLC sinogram and jaw pattern. MLC corrections were applied based on the beams eye view of the target motion, which is dependent on gantry angle and is consistent with the behavior during treatment. For example, if the gantry is at 0 degree (pointing down), motion in the IEC‐*X* direction is corrected, but motion in IEC‐*Z* is not. An example of applying motion corrections to the MLC sinogram is shown in Figure 3. The MLC leaf openings for each of the 10 plans were modified based on the location of the target in each phase relative to the reference phase. For subject 3, the target is displaced by less than 3 mm (approximately half the projected MLC width at isocenter) in the axial plane for phases of inhale 20% through exhale 80%; therefore, the MLC sinogram is not modified as the motion is not large enough to warrant a shift in MLC leaves.

Lastly, the jaw positions for each of the 10 plans were modified based on the location of the target in each phase, similar to the MLC compensation. In Figure 3, the jaw settings are at the centered position for the inhale 40% phase as this was chosen as the reference phase for this subject. The jaw offsets in Figure 3B correspond to the target IEC‐*Y* positions from Figure 1. The jaws are stationary throughout treatment for a given phase for computation purposes as the target occupies one location in that phase of the 4DCT, and the 1‐cm jaw setting was used for this example. Plans with dynamic jaws (sometimes referred to as “running start and stop”) will have more complicated jaw motion at the beginning and end of the treatment, but dynamic jaws cannot be used for 1‐cm jaw plans.

The plans were calculated on the corresponding 4DCT phases. The calculations were performed with the tomotherapy convolution/superposition (C/S) algorithm using the same beam model as the clinical dose calculations. Dose was calculated at each sub‐projection, or 153 discrete projection angles per gantry rotation.^2^, ^19^ The size of the dose grid was 2 mm or smaller in each direction.

### 2.5 Deformable image registration

Once doses were calculated, DIR was used to transfer the dose from each phase of the 4DCT to the reference phase. For the example cases in this work, DIR was performed in MIM, which uses an intensity‐based free‐form deformation algorithm with a sum of squared differences as a similarity measurement.^20^ The resolution of all 4DCTs was 1 × 2 × 1 mm^3^ or smaller and each registration for all subjects was reviewed and refined with the *Reg Reveal* and *Reg Refine* tools in MIM, which allow the user to modify and improve the quality of the registration in different regions.^10^

The recommendations of Task Group 132 should be followed to verify the accuracy of the DIR, which includes commissioning/verification of the algorithm and patient‐specific verification of each registration.^21^ The MIM DIR algorithm used for the examples in this work has been verified in the literature.^10^, ^20^, ^22^, ^23^ Azcona et al. validated DIR in MIM using the “point‐validated pixel‐based breathing thorax” (POPI) model.^10^ The mean 3D target registration error ranged from 0.6 to 1.5 mm among 500 landmarks, indicating that the registrations were accurate considering the resolution of the images. Guy et al. found that contours transferred between 4DCT phases by MIM were acceptable or only requiring minor revisions, by a physician, 91.8% of the time.^22^ In addition, the accuracy of the MIM DIR algorithm and the resulting dosimetric accuracy was validated by Mittauer et al.^23^

In addition, several steps were taken to verify the accuracy of the DIR for each example case.^24^ For each registration, the transformed image was visually compared to the reference image by inverting the transformed image in gray scale and fusing with the reference image, providing a difference fusion.^24^ A script was written to perform all nine fusions and display them together such that visual inspection was quick. This step is intended to identify obvious errors in registration. Next, the GTV contour was transferred from the reference phase and verified that the boundaries are within 1–2 voxels of the visible solid tumor on each phase.^24^ Next, the histogram of Jacobian determinant values was analyzed for each transformation to ensure that the registrations were valid (no negative values indicating ripping, folding, or tearing). Lastly, Pearson correlation coefficients were calculated for each registration and verified to be greater than 0.90.

### 2.6 Dose accumulation and plan analysis

Once all DIR transformations had been verified and doses had been transferred to the reference phase, the doses were summed, resulting in the Synchrony‐accumulated dose. Prior to calculation, the Radixact couch was added to each phase of the 4DCT to match the position of the couch on the planning image, which was performed using the MATLAB. The dose was scaled by the number of fractions to result in the total dose distribution, which assumes that the patient breathes the same and the dose is delivered the same each fraction. All results in this work were for the full prescription and not individual fractions.

Lastly, the planned and Synchrony‐accumulated doses were compared. This is analogous to comparing the planned and delivered doses, where the Synchrony‐accumulated dose is the best approximation of the dose delivered with tracking active. For the examples in this work, the doses were compared using differences in OAR dose metrics typically used for lung and liver SBRT plan evaluation at our institution.^15^, ^16^ If the motion compensation step is skipped, the result of the workflow would result in a “planned accumulated” dose. This represents an estimate of what would be delivered to the patient if the motion were ignored, and the static plan were delivered with the patient breathing freely. However, this work did not investigate the impact of not using motion management on tomotherapy treatments as this has been done extensively in the literature.^1^, ^2^, ^4^, ^25^, ^26^, ^27^, ^28^

To validate the accuracy of the dose calculation and accumulation, a test case was performed following all the same steps of the framework but using the reference phase image for each of the 10 images in the framework instead of the original 10 phases of the 4DCT with varying anatomy. The accumulated dose distribution was compared to the planned dose and no dose difference was found, which provided confidence in the accuracy of the framework.

## 3 RESULTS

Table 2 displays a summary of the results of generating the motion‐synchronized treatment plans. The jaw sway range refers to the field position at isocenter and corresponds to the IEC‐*Y* motion of the tumor. The maximum range the jaws can mechanically move is 2.5 cm for the 2.5‐cm jaw setting and 4 cm for the 1‐cm jaw setting. The percent of leaf openings modified was calculated by comparing the pre‐synchronized sinograms to the synchronized sinograms. Only nonzero leaf openings in the pre‐synchronized sinogram are considered in the calculation. If the percent of leaf openings modified is 10%, it indicates that for 90% of the leaf openings (and approximately 90% of the time), there was insufficient motion in the axial plane to warrant MLC shifts. The most MLC leaf shifts occurred for Lung 3, despite not having the largest amplitudes in *X* and *Z*. This is because this value depends not only on the range of motion in *X* and *Z*, but also the relative centering of the target in the reference phase. If the target is not in the center of the range of motion in the axial plane in the reference phase, the target will spend more time off‐axis and more leaf openings will be modified than if centered. Overall, jaw motion accounts for the majority of the changes to the treatment plan as most patients (and all patients in this work) have a majority of motion in the *Y* direction.

**TABLE 2:** Peak‐to‐peak tumor motion observed on the 4DCT for each subject and resulting jaw and MLC changes to the treatment plans

Subject	Amplitude (mm) *X*, *Y*, *Z*	Jaw setting (cm)	Jaw sway range (cm)	% Leaf openings modified
Lung 1	4, 19, 5	2.5	1.89	2.5
Lung 2	2, 12, 7	2.5	1.22	9.0
Lung 3	3, 28, 6	1	2.78	13.7
Lung 4	4, 25, 7	2.5	2.49	4.4
Lung 5	2, 30, 5	1	3.07	6.5
Liver 1	3, 5, 5	2.5	0.54	7.1
Liver 2	2, 13, 3	1	1.30	0.0
Liver 3	1, 6, 6	2.5	0.62	5.8
Liver 4	2, 10, 3	2.5	0.98	0.0
Liver 5	2, 9, 4	1	0.95	8.0
Ave	3, 15, 5	–	1.60	5.7

Abbreviations: 4DCT, four‐dimensional computed tomography; MLC, multi‐leaf collimator.

Tables 3 and 4 display the planned dose and dose deviations between the Synchrony‐accumulated dose and the planned dose for the lung and liver cases, respectively. The dose deviations are displayed three ways: in gray, as a percent of the planned dose, and as a percent of the dose objective. For example, for Lung 1 the planned dose to the Normal 2 cm D0.03 cm^3^ was 20.0 Gy and the Synchrony‐accumulated dose was 18.1 Gy. Therefore, the dose deviation is −1.9 Gy, which is −9.4% compared to the planned dose of 20.0 Gy or −4.0% compared to the objective of 47.0 Gy.

**TABLE 3:** Dose difference statistics (Synchrony accumulation minus plan) for the lung subjects evaluated using common lung SBRT metrics

		Normal 2 cm D0.03 cm^3^	Skin D1 cm^3^	Lungs‐GTV D15%	Cord D0.03 cm^3^	Chest wall D0.1 cm^3^	Esophagus D0.1 cm^3^	Heart D0.1 cm^3^	PTV D98%	GTV D95%	Body max dose diff	Body min dose diff
Dose objective (Gy)		47.0	32.0	12.5	22.0	44.0^a^	30.0^a^	50.0	50.0	60.0		
Planned dose (Gy)	Lung 1	20.0	10.1	3.6	14.6	52.3^A^	10.1	8.0	47.9	63.9		
	Lung 2	46.5^B^	31.8	10.6	21.0^C^	51.8	35.7	7.0	49.4	63.3		
	Lung 3	25.4	13.9	6.9	10.3	37.8	9.3	9.7	51.5	63.9		
	Lung 4	23.7	8.2	8.9	10.0	14.1	16.1	28.3	50.5	67.9		
	Lung 5	28.3	15.7	5.7	16.4	35.6	11.6	11.5	50.5	70.6		
Difference relative to planned (Gy)	Lung 1	−1.9	−1.5	−0.1	−1.2	**−1.7** ^A^	−0.5	−0.3	**−1.7**	−1.3	**9.7**	**−6.9**
	Lung 2	**−2.3^B^**	−0.4	0.0	**2.2** ^C^	−1.1	−0.9	**−2.7**	−0.9	−0.1	**10.5**	**−6.2**
	Lung 3	−1.2	**−1.9**	0.1	−1.0	**−2.3**	−0.9	0.1	−3.0	**−1.5**	**11.5**	**−10.4**
	Lung 4	−1.2	−1.1	−0.9	−1.0	**−1.5**	**−1.5**	0.2	**−6.5**	**−2.9**	**7.3**	**−14.7**
	Lung 5	**−3.3**	**−3.8**	−0.6	**−3.6**	**−3.7**	1.0	−0.1	**−5.0**	**−1.9**	**13.0**	**−12.0**
Difference relative to planned (%)	Lung 1	**−9.4**	**−14.7**	−3.1	**−8.1**	−3.3	**−5.0**	−4.0	−3.5	−2.1		
	Lung 2	−4.9	−1.4	0.5	**10.6**	−2.1	−2.4	**−39.1**	−1.9	−0.2		
	Lung 3	−4.6	**−13.3**	2.2	**−10.0**	**−6.0**	**−9.7**	1.5	**−5.8**	−2.3		
	Lung 4	−4.9	**−13.9**	**−9.8**	**−9.6**	**−10.3**	**−9.4**	0.6	**−12.9**	−4.2		
	Lung 5	**−11.6**	**−24.0**	**−10.4**	**−22.0**	**−10.4**	**8.3**	−0.6	**−9.9**	−2.6		
Difference relative to objective (%)	Lung 1	−4.0	−4.7	−0.9	**−5.4**	−3.9	−1.7	−0.6	−3.4	−2.2		
	Lung 2	−4.9	−1.3	0.4	**10.1**	−2.5	−2.9	**−5.5**	−1.9	−0.2		
	Lung 3	−2.5	**−5.8**	1.2	−4.7	**−5.2**	−3.0	0.3	**−6.0**	−2.4		
	Lung 4	−2.5	−3.5	**−7.0**	−4.4	−3.3	**−5.0**	0.3	**−13.1**	−4.8		
	Lung 5	**−7.0**	**−11.8**	−4.7	**−16.4**	**−8.4**	3.2	−0.1	**−10.0**	−3.1		

Values are bolded if the absolute difference is larger than a 1.5 Gy or 5%. Superscript letters refer to examples in Section 4.

Abbreviation: SBRT, stereotactic body radiation therapy.

^a^May extend up to 52.5 Gy if overlap with the PTV.

**TABLE 4:** Dose difference statistics (Synchrony accumulation minus plan) for the liver subjects evaluated using common liver SBRT metrics

		Chest wall D0.1 cm^3^	Liver‐GTV D700 cm^3^	Bowel D0.5 cm^3^	Stomach D0.5 cm^3^	Heart D0.1 cm^3^	Kidneys D33%	PTV D95%	GTV D95%	Body max dose diff	Body min dose diff
Dose objective (Gy)		44.0^a^	15.0	30.0	30.0	50.0	15.0	^b^	^b^		
Planned dose (Gy)	Liver 1	40.0	8.7	29.0^D^	19.9	1.2	0.7	38.7	44.6		
	Liver 2	27.0	1.8	7.4	9.9	0.6	4.6	41.2	41.3		
	Liver 3	22.9	13.8	1.2	16.0	49.2	0.3	57.4	61.0		
	Liver 4	49.3	9.3	1.0	7.5	13.6	0.3	49.4	52.2		
	Liver 5	40.1	1.1	29.4^E^	9.2	0.6	6.6	55.0	56.5		
Difference relative to planned (Gy)	Liver 1	−0.2	−0.2	**5.9** ^D^	−0.9	0.0	0.1	−1.1	−0.2	**12.5**	**−6.7**
	Liver 2	−1.2	0.2	0.1	0.1	0.0	−0.1	−0.3	0.4	**7.3**	**−7.2**
	Liver 3	−0.3	0.2	0.1	0.0	**1.5**	0.0	−0.2	−0.3	**6.4**	**−3.8**
	Liver 4	**5.3**	−0.3	0.0	0.0	−0.6	0.0	**−1.8**	0.5	**11.4**	**−12.2**
	Liver 5	−0.4	0.4	**−4.0** ^E^	0.3	0.0	0.2	0.2	0.7	**19.8**	**−9.5**
Difference relative to planned (%)	Liver 1	−0.6	−1.8	**20.2**	−4.4	1.6	**9.5**	−2.8	−0.4		
	Liver 2	−4.6	**11.9**	1.5	0.5	−1.8	−1.5	−0.7	1.0		
	Liver 3	−1.5	1.4	4.3	−0.2	3.1	0.0	−0.3	−0.5		
	Liver 4	**10.7**	−2.8	−1.0	−0.5	−4.4	−2.9	−3.6	1.0		
	Liver 5	−0.9	**36.8**	**−13.5**	2.7	**6.8**	2.6	0.4	1.2		
Difference relative to objective (%)	Liver 1	−0.5	−1.1	**19.5**	−2.9	0.0	0.5	−2.7	−0.4		
	Liver 2	−2.8	1.4	0.4	0.2	0.0	−0.5	−0.7	1.0		
	Liver 3	−0.8	1.3	0.2	−0.1	3.1	0.0	−0.3	−0.5		
	Liver 4	**12.0**	−1.7	0.0	−0.1	−1.2	−0.1	−3.6	1.0		
	Liver 5	−0.9	2.8	**−13.3**	0.8	0.1	1.1	0.5	1.3		

Values are bolded if the absolute difference is larger than a 1.5 Gy or 5%. Superscript letters refer to examples in Section 4.

Abbreviation: SBRT, stereotactic body radiation therapy.

^a^ May extend up to 52.5 Gy if overlap with the PTV.

^b^ The prescription ranges from 40 to 55 Gy.

For the lung subjects, 21 of 35 OAR dose differences were greater than 5% deviation compared to the plan dose. When the difference is compared to the objective dose, 11 of 35 OAR dose differences were greater than 5% deviation. For the liver subjects, 7 of 30 OAR dose differences compared to planned were greater than 5% deviation and 3 of 30 OAR dose differences compared to the objective were greater than 5% deviation.

Body maximum dose differences were tabulated as an indication of the magnitude of differences that were observed in the body, which may not be represented in a single slice of the dose difference distribution or in the volumetric dose metrics. The maximum dose difference in the body was on average 10.9 ± 3.9 Gy among the 10 subjects and the minimum difference (most negative) in the body was on average −9.0 ± 3.4 Gy. The locations of the differences are generally in anatomy that is in field for one plan but out of field for the other. These values indicate how OAR doses may be much larger or smaller than planned for any given subject. The largest positive dose deviation for any subject was 19.8 Gy for Liver 5, which occurred inside healthy liver tissue. The largest negative dose deviation for any subject was −14.7 Gy for Lung 4, which occurred inside healthy lung tissue.

Although investigating target dose was not the goal of this work, the GTV and PTV doses were included in Tables 3 and 4 to demonstrate that target dose is maintained between the planned and synchronized treatments. The average dose difference from planned among the 10 subjects was −2.0 ± 2.2 Gy for the PTV and −0.7 ± 1.2 Gy for the GTV.

In general, there was a decrease in maximum dose metrics for the Synchrony accumulation compared to the plan. This is especially true for structures that are relatively static compared to the tumor such as the skin, spinal cord, chest wall, esophagus, and heart. The average dose difference among all subjects and all max dose metrics (dose to 1 cm^3^ or less) for these structures was −5.6% relative to the plan and −2.5% relative to the objective. This is caused by the synchronization spreading out the radiation field, therefore blurring the dose to static structures and reducing maximum doses.

**FIGURE 4:**
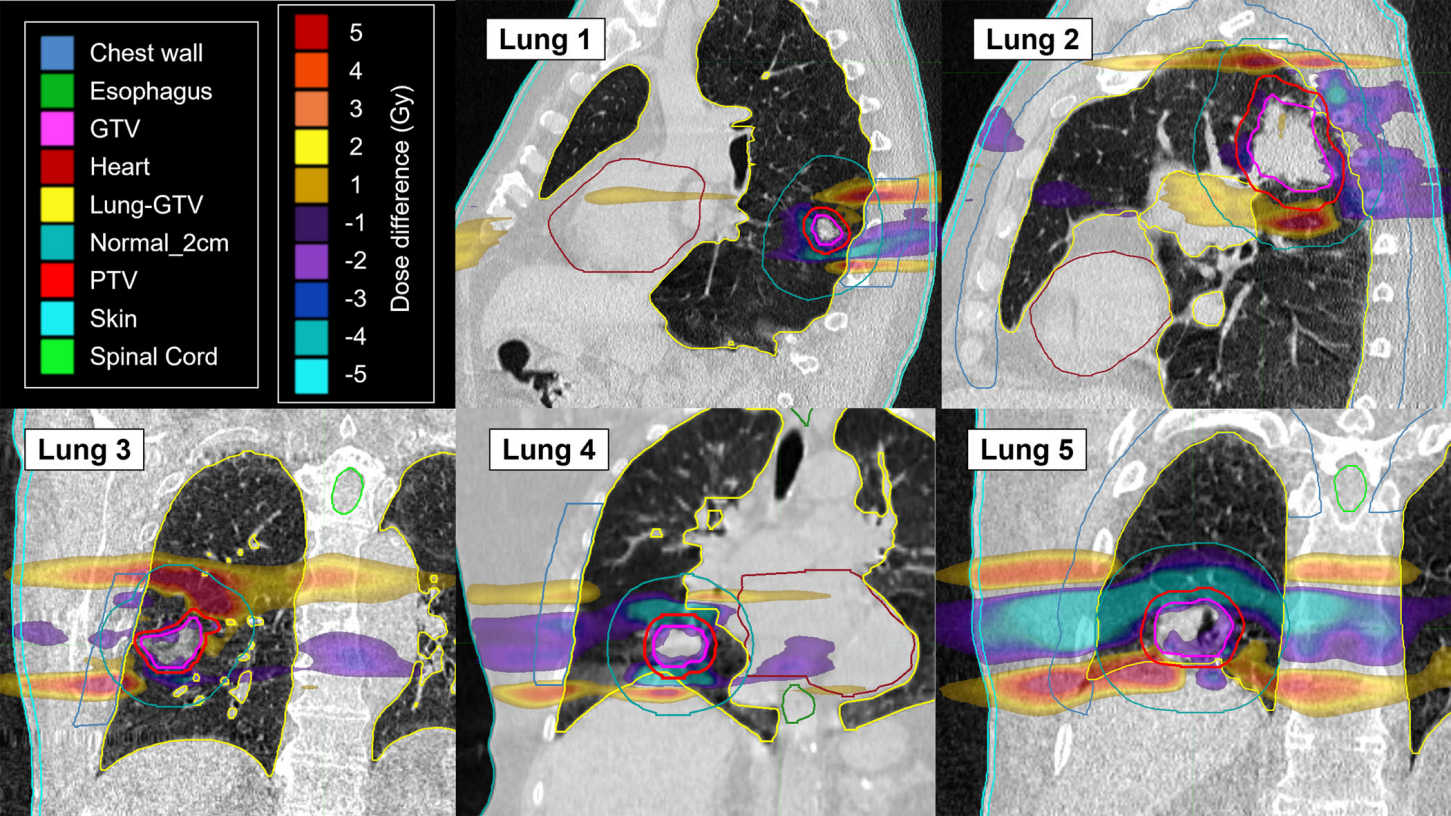
Dose differences (Synchrony accumulation minus plan) for each lung subject for the full prescription. For example, a 5‐Gy dose difference is a 10% difference relative to the prescription of 50 Gy

**FIGURE 5:**
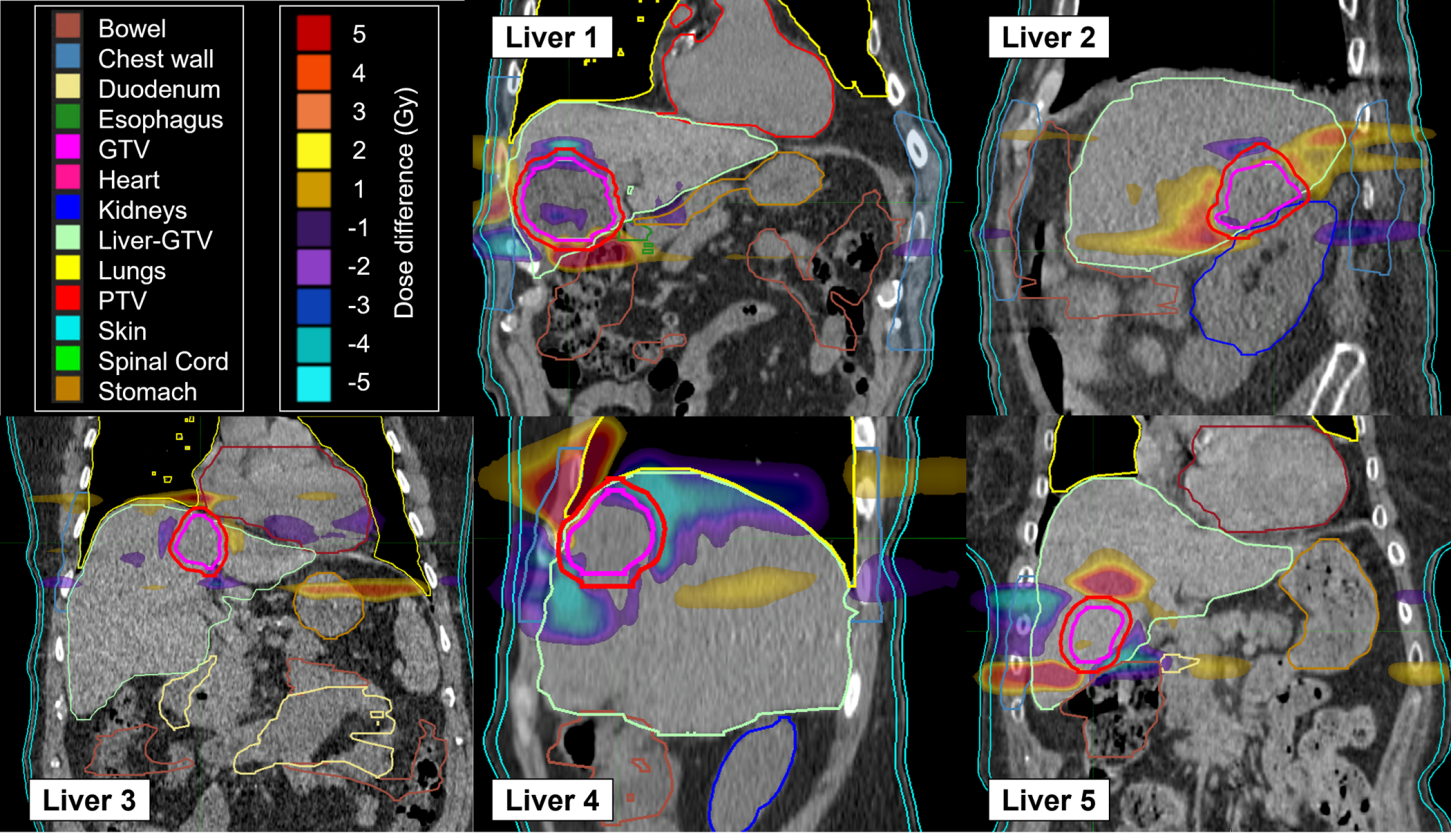
Dose differences (Synchrony accumulation minus plan) for each liver subject for the full prescription. For example, a 5‐Gy dose difference is a 10% difference relative to a prescription of 50 Gy

Figures 4 and 5 display dose distributions of the difference between the planned and Synchrony accumulation doses for the lung and liver subjects, respectively. The dose differences are small within the GTV, indicating that the motion synchronization is achieving the goal of keeping the dose to the target after synchronization the same as planned. Due to jaw sway, the dose differences tend to be negative (lower dose for synchronized) in stationary anatomy in the same transverse slice as the tumor in the reference plan. Alternatively, the dose differences tend to be positive at static anatomical locations inferior or superior to the target. This can be observed along the spinal column and chest wall for the lung cases in Figure 4.

The dose distributions reveal dose differences that may not be represented by the treatment evaluation metrics in Tables 3 and 4. For example, the largest absolute deviation for any OAR metric in Tables 3 and 4 was 6.5 Gy, but several of the cases had absolute dose deviations of larger than 10 Gy somewhere in the body. The dose difference plots provide information on the locations of the dose differences and their volume within the subject.

## 4 DISCUSSION

This work revealed scenarios where knowledge of the dose deviations may be useful in making clinical decisions. Several of these scenarios are presented in the superscript letters in Tables 3 and 4. For example, in Example A the D0.1 cm^3^ to the chest wall for Lung 1 was 52.3 Gy, just below the objective of 52.5 Gy. However, the synchronized plan reduces that dose to 50.6 Gy, which is not as close to the objective. Similarly in Example B, the D0.03 cm^3^ for Lung 2 to the Normal 2 cm is 46.5 Gy for the plan, the objective is 47 Gy, and the synchronized plan decreases the value to 44.2 Gy. In these examples, the dose backed off a critical constraint and made the treatment plan more acceptable. Conversely, in Example C the D0.03 cm^3^ to the spinal cord for Lung 2 increased from 21.0 Gy for the plan, past the limit of 22.0 Gy, to 23.2 Gy for the synchronized treatment. In this example, the dose increased enough to make the plan fail one of the constraints that was not failing before.

Examples D and E for the liver cases are additional scenarios where knowledge of the dose deviations may increase or decrease acceptability of a plan. In Example D for Liver 1, the bowel D0.5 cm^3^ was 29.0 Gy for the plan, 30 Gy for the objective, and 34.9 Gy for the synchronized delivery. Therefore, the objective would be violated from the synchronized delivery. However, the opposite was observed in Example E for Liver 4, where the bowel D0.5 cm^3^ was 29.4 Gy for the plan, 30 Gy for the objective, and 25.4 Gy for the synchronized delivery. In this case, the plan would achieve the bowel objective much easier with the synchronized delivery than planned. This could allow for greater emphasis on tumor coverage in subsequent fractions.

The results of this work are intended to be examples of the magnitude of OAR dose deviations that can be observed from tracking treatments and how these calculations could potentially sway clinical decisions, both in the initial planning and in an online adaptive planning process. The OAR dose deviations are expected to be representative of deviations that might be observed from other tracking solutions that do not consider the location changes of OARs (e.g., CyberKnife Synchrony or the Kilovoltage Intrafraction Monitoring system).^29^ Whether an institution uses the dose deviation information from these calculations to make clinical decisions is up to the institution as no formal recommendation can be made from this work.

One may attempt to predict the location of dose deviations to OARs due to respiration provided enough spatial information is known. If the displacement between the target and an OAR is nonrigid, that is, the distance between the two changes as a function of respiratory phase, the OAR dose may change from the tracked treatment. For example, if the static plan is generated at the end of exhale phase and the target is in the middle of the liver, one can expect the primary beam to be directed more inferior than planned because the target will spend more time inferior due to motion of the diaphragm pushing the liver and tumor inferior. Static structures inferior to the transverse locations of the target in the reference phase may receive higher dose than planned and static structures superior may receive less dose than planned. This warrants careful choice of the reference phase. If the reference phase is mid‐ventilation, the target will move in both directions and if the reference phase is at end of inhale or end of exhale, the target will only move in one direction relative to the reference phase. However, one must know how the OAR moves in relation to the tumor. Also, predicting the *magnitude* of dose differences is more complex and necessitates the accumulation calculations.

The results showed that the dose to the GTV is maintained more closely than the PTV, which is expected as the purpose of the PTV is to expand the GTV to account for uncertainties and ensure the prescription is delivered to the GTV.^30^ Underdosings of the target (1% for GTV and ∼4% for PTV) have been reported for other studies comparing 4D‐accumulated, motion‐compensated tracking doses to planned dose.^10^, ^31^ The differences in target dose may be from the dose deformation effect,^11^, ^31^, ^32^, ^33^ the unflattened profile of the Radixact beam (causing maximum decrease in output of 3%–4% for the 1‐cm jaw and 1%–2% for the 2.5‐cm jaw as the target moves off‐axis),^3^, ^7^ the discrete nature of MLC compensation (which allows for axial motion less than ±3.125 cm to be uncompensated), and uncertainties in the framework. These effects cause changes to the dose distribution *within* the field and penumbra, which are much smaller than changes to the dose distribution due to changes *to* the field (such as jaw sway).

The first uncertainty that should be considered is the uncertainty of the 4DCT itself. These datasets are often noisy, which can cause differences in radiological pathlengths even if the anatomy is perfectly static. Artifacts are also common due to discrete binning of projections and the breathing motion,^24^ which will cause beam perturbations. In addition, the DIR is affected by the resolution and accuracy of the 4DCT images. DIR registrations may have offsets of 1–2 voxels,^21^, ^24^ which is between 1 and 4 mm for the 4DCT images used in this work. The recommendations of TG‐132 should be used to check the accuracy of 4DCT images and DIR used for these calculations.^24^ In addition, there is uncertainty in the determination of the 3D target position in each 4DCT phase. The centroid of the GTV is used as the target position for the cases in this work. If there are nonrigid deformations of the target, the rigid motion assumption breaks down and the centroid cannot accurately describe the motion. However, Synchrony always assumes that target motion is rigid; therefore, this uncertainty is always present in these treatments. Lastly, there is uncertainty in the tomotherapy (C/S) dose calculation algorithm, especially in heterogenous regions. However, Sterpin et al. found good agreement between the tomotherapy C/S algorithm and Monte Carlo calculations for most lung cases.^34^ In addition, the C/S algorithm is validated and routinely used for clinical lung treatments. Overall, changes in the dose distribution caused by changes in the direction of the primary beam are expected to be much larger than changes in the dose distribution caused by uncertainties in dose calculation, DIR, and 4DCT acquisition.

It is critical that the patient is aligned before treatment at the same phase of respiration that was used as the reference 4DCT phase for the calculations. For example, if the patient will be aligned before the treatment using a maximum inhalation breath hold scan, then the maximum inhale 4DCT phase should be used as the reference phase for the calculations. If a different phase is used, then the calculations may not be as representative of dose deviations that can be expected. Also, the patient may breathe differently during the treatment than during the 4DCT simulation. However, the 4DCT is the most commonly applied approximation of how the patient will breathe during the treatment. Breath‐coaching could be used to minimize this difference. It is not expected that the phase and shape of the patient's respiration will have a large effect on the overall dose distribution as the target is still limited to 10 locations based on the 4DCT data and therefore the shape of respiration only adjusts the amount of time spent in each of the 10 phases. Overall, the calculations should be viewed as the best approximation of what will be delivered to the patient from the tracked treatment and not the exact dose delivery.

## 5 CONCLUSIONS

Dose distributions calculated for tracking treatments assume that OARs move rigidly with the target. In this work, we developed an in‐house dose accumulation framework to quantify the inaccuracy introduced by that assumption for a subset of clinical examples of nonrigid body deformation. Doses were reduced in static OARs within the same transverse slice as the target observed in its reference phase. This is the consequence of allowing the jaw to sway to track the sup/inf motion of the target. This also caused doses to static OARs superior or inferior to the transverse slice of the target to increase as tracking may make them in field for a portion of the treatment. Lastly, differences in OAR doses between the planned and the synchronized deliveries can be leveraged during the treatment planning process. This could be done prior to the initial treatment fraction based on population statistics for motion models, during online adaptive radiation therapy, and/or offline adaptation based on estimates of accumulated dose. Target dose remained relatively constant, confirming that the goal of tracking was achieved. The current work demonstrates that OAR dose deviations should be considered for real‐time tracking systems in addition to maintaining target dose, which has been the focus of most measurements and investigations prior to this work.

## CONFLICT OF INTEREST

John E. Bayouth has ownership interest in MR Guidance, LLC, which has business activity with a company that utilizes image guided radiation therapy technology (ViewRay, Inc.). Although this project was not sponsored externally, the data was collected on a Radixact system (Accuray Inc.) provided to UW–Madison under a research agreement (Bayouth, PI). Randall Kimple is supported in part by the University of Wisconsin Carbone Cancer Center Support Grant (No. P30 CA014520). The remaining authors have no conflicts of interest to disclose.

## AUTHOR CONTRIBUTIONS

The authors confirm contribution to the paper as follows: study conception and design: Ferris WS, Bayouth JE, Chao EH; data collection: Ferris WS; analysis and interpretation of the results: all authors; draft manuscript preparation: Ferris WS; all authors reviewed the results and approved the final version of the manuscript.

## Supporting information

Supporting Information

## Data Availability

Data are available upon reasonable request to the authors and approval of UW Health IRB.
