# Supplementary material for: Using 4D dose accumulation to calculate organ‐at‐risk dose deviations from motion‐synchronized liver and lung tomotherapy treatments
Source: J Appl Clin Med Phys. 2022 Apr 29;23(7):e13627. doi: 10.1002/acm2.13627 (PMC9278681; doi:10.1002/acm2.13627)
Supplement: Supplementary file 1 — Supporting Information [file ACM2-23-e13627-s001.docx]

Using 4D Dose Accumulation to Calculate Organ at Risk Dose Deviations from Motion-Synchronized Liver and Lung Tomotherapy Treatments

William S. Ferris^1^­, Edward H. Chao^2^, Jennifer B. Smilowitz^1,3^, Randall J. Kimple^1,3,4^, John E. Bayouth^3^, and Wesley S. Culberson^1^

*^1^ Department of Medical Physics, School of Medicine and Public Health, University of Wisconsin-Madison, Madison, WI 53705*

*^2^ Accuray Inc., Madison, WI 53717*

*^3^ Department of Human Oncology, School of Medicine and Public Health, University of Wisconsin-Madison, Madison, WI 53792*

*^4^ University of Wisconsin Carbone Cancer Center, University of Wisconsin-Madison, Madison, WI 53792*

Corresponding Author:

*William S. Ferris, M.S.*

*Department of Medical Physics*

*School of Medicine and Public Health*

*University of Wisconsin-Madison*

*1111 Highland Avenue, Madison, WI 53705*

*Tel.: (402) 889-3894*

*Email: williamferris0@gmail.com*

Running title: “OAR dose deviations from tracking treatments”

Keywords: *Tomotherapy, Radixact, Synchrony*

Author Contribution Statement

The authors confirm contribution to the paper as follows: study conception and design: Ferris WS, Bayouth JE, Chao EH; data collection: Ferris WS; analysis and interpretation of the results: all authors; draft manuscript preparation: Ferris WS; all authors reviewed the results and approved the final version of the manuscript.

Acknowledgements

The authors thank the students and staff of the UWMRRC for their continued support, the UWRCL and UWADCL customers whose calibrations help support ongoing student research at the UWMRRC. We also thank Accuray, Inc. for their technical support in this work.

Conflict of Interest

John E. Bayouth has ownership interest in MR Guidance, LLC, which has business activity with a company that utilizes image guided radiation therapy technology (ViewRay, Inc.). While this project was not sponsored externally, the data was collected on a Radixact system (Accuray, Inc.) provided to UW-Madison under a research agreement (Bayouth, PI).

Randall Kimple is supported in part by the University of Wisconsin Carbone Cancer Center Support Grant (P30 CA014520).

The remaining authors have no conflicts of interest to disclose.

Data Availability Statement

Data are available upon reasonable request to the authors and approval of UW Health IRB
